# Synthesis of Ferrofluids Made of Iron Oxide Nanoflowers: Interplay between Carrier Fluid and Magnetic Properties

**DOI:** 10.3390/nano7110373

**Published:** 2017-11-05

**Authors:** Federico Spizzo, Paolo Sgarbossa, Elisabetta Sieni, Alessandra Semenzato, Fabrizio Dughiero, Michele Forzan, Roberta Bertani, Lucia Del Bianco

**Affiliations:** 1 Dipartimento di Fisica e Scienze della Terra, Università di Ferrara, I- 44122 Ferrara, Italy; federico.spizzo@unife.it; 2 Dipartimento di Ingegneria Industriale, Università di Padova, I- 35131 Padova, Italy; paolo.sgarbossa@unipd.it (P.S.); elisabetta.sieni@unipd.it (E.S.); fabrizio.dughiero@unipd.it (F.D.); michele.forzan@unipd.it (M.F.); roberta.bertani@unipd.it (R.B.); 3 Dipartimento di Scienze del Farmaco, Università di Padova, I- 35131 Padova, Italy; alessandra.semenzato@unipd.it

**Keywords:** magnetic nanoparticles, ferrofluids, thermal decomposition, nanoflower structure, spinel iron oxides, magnetic interactions, Mössbauer spectroscopy, magnetic heating, spin canting, superparamagnetism

## Abstract

Ferrofluids are nanomaterials consisting of magnetic nanoparticles that are dispersed in a carrier fluid. Their physical properties, and hence their field of application are determined by intertwined compositional, structural, and magnetic characteristics, including interparticle magnetic interactions. Magnetic nanoparticles were prepared by thermal decomposition of iron(III) chloride hexahydrate (FeCl_3_·6H_2_O) in 2-pyrrolidone, and were then dispersed in two different fluids, water and polyethylene glycol 400 (PEG). A number of experimental techniques (especially, transmission electron microscopy, Mössbauer spectroscopy and superconducting quantum interference device (SQUID) magnetometry) were employed to study both the as-prepared nanoparticles and the ferrofluids. We show that, with the adopted synthesis parameters of temperature and FeCl_3_ relative concentration, nanoparticles are obtained that mainly consist of maghemite and present a high degree of structural disorder and strong spin canting, resulting in a low saturation magnetization (~45 emu/g). A remarkable feature is that the nanoparticles, ultimately due to the presence of 2-pyrrolidone at their surface, are arranged in nanoflower-shape structures, which are substantially stable in water and tend to disaggregate in PEG. The different arrangement of the nanoparticles in the two fluids implies a different strength of dipolar magnetic interactions, as revealed by the analysis of their magnetothermal behavior. The comparison between the magnetic heating capacities of the two ferrofluids demonstrates the possibility of tailoring the performances of the produced nanoparticles by exploiting the interplay with the carrier fluid.

## 1. Intruduction

Magnetic nanofluids, also indicated as ferrofluids, consist of colloidal magnetic nanoparticles (with size between 1 and 100 nm) that are dispersed in a carrier fluid. They are extremely versatile nanomaterials, showing both liquid and magnetic properties, with a huge potential in a large number of technological sectors, such as electric and thermal engineering, electronics, magneto-optics, catalysis, waste water treatment [1,2,3,4,5]. Moreover, ferrofluids have been attracting an increasing interest for biomedical applications since the magnetic nanoparticles may act as contrast media in magnetic resonance imaging, tags for magnetic biosensing, drug delivery carriers, magnetic hyperthermia agents for cancer therapy or/and thermally activated drug release [6,7,8,9,10,11]. The properties of the ferrofluids, and, hence, their field of application strictly depend on the features of the magnetic nanoparticles, such as the chemical composition (metallic ferromagnets and their oxides), the size, the shape and the crystallinity. In turn, these characteristics are strictly connected to the saturation magnetization and the magnetic anisotropy. Moreover, the concentration and the colloidal stability of the nanoparticles in the carrier fluid are crucial.

Strong research efforts have been carried out during the last decade to develop novel synthetic routes that are aimed at controlling these different parameters [12,13,14,15]. Particular attention has been directed to the synthesis of nanoparticles of spinel iron oxides (i.e., magnetite and maghemite), which are very suitable materials for biomedical applications due to their high biocompatibility. For this purpose, the alkaline co-precipitation of ferrous and ferric salts in aqueous media and the thermal decomposition of organometallic compounds are among the most widely used methods [12]. Generally, the latter route includes the use of high boiling point organic solvents, which can also act as surfactants. The surfactants stabilize the colloidal nanoparticles and impede the direct contact among the nanoparticles and, hence, the magnetic exchange interaction. However, they cannot completely hinder the onset of dipolar interparticle magnetic interactions, which, as it is now quite well established, may markedly modify the magnetothermal behavior of an assembly of nanoparticles, as compared to the case of non-interacting particles. In fact, isolated nanoparticles can show superparamagnetic relaxation when their thermal energy is comparable to the anisotropy energy barrier for moment reversal [16], a parameter proportional to the particle volume. Magnetic interactions lead to an increase of the anisotropy energy barriers, which become interdependent, so that the particle moments do not relax independently any longer [16,17]. Hence, dipolar interactions may represent an additional degree of freedom in order to govern the properties of ferrofluids, even if, in turn, they depend on the particle magnetization, the size of the nanoparticles and their interdistance. Hence, the synthesis route, the compositional, structural, and magnetic properties of the nanoparticles, as well as their local spatial arrangement and the degree of magnetic coupling are all strictly intertwined factors. In order to modify just the strength of the magnetic dipolar interactions of an assembly of a given type of nanoparticles, their concentration in the dispersing medium is usually varied [16,18].

In this context, we report about the production and the compositional, structural, and magnetic characterization of iron oxide nanoparticles, subsequently used in the preparation of ferrofluids. The synthesis was accomplished by thermal decomposition using hydrated ferric chloride as iron precursor and strong polar 2-pyrrolidone [19], which acted both as surfactant and solvent. The predicted advantage of this method is that 2-pyrrolidone allows the nanoparticles to be dispersed in polar fluids without the need of any further functionalization [20,21]. In our case, the produced nanoparticles were dispersed in two different carrier fluids, water and polyethylene glycol 400 (PEG), which are commonly used to prepare isotonic solutions for in vitro biological experiments. We will show that the produced nanoparticles are arranged in compact structures, resembling the shape of a nanoflower, which are substantially stable in water, whereas in PEG they tend to disaggregate. Hence, the interplay with the carrier fluid can be used as a tool to modulate the aggregation state of the nanoparticles and, hence, the strength of the interparticle magnetic interactions, thus conferring to the ferrofluids different properties, as shown with regard to the magnetic heating capacity.

## 2. Experiment

### 2.1. Synthesis of the Nanoparticles and of the Ferrofluids

Iron oxide nanoparticles were prepared by thermal decomposition of iron(III) chloride, according to the method proposed by Li et al. [19]; FeCl_3_·6H_2_O, 2-pyrrolidone and all of the solvents that were used in the synthesis were purchased from Sigma-Aldrich and were used without further purification.

In detail, a solution of iron(III) chloride hexahydrate was prepared by dissolving 2.70 g of iron salt in 50 mL of 2-pyrrolidone (relative concentration 5.40 × 10^−2^ g/mL) in a round bottomed flask mounted with a reflux condenser. The solution was purged with nitrogen in a Schlenk line and heated by mean of a heating mantle from 25 to 315 °C at 10 °C/min and was kept at 315 °C for 24 h under vigorous magnetic stirring in reflux conditions. After the solution was cooled down to room temperature, 10 mL of methanol were added and the produced nanoparticles precipitated in a threefold volume of diethyl ether. The precipitate was separated by means of a permanent magnet and purified by repeated (four times) dispersion in methanol (10 mL) and precipitation with acetone (200 mL). The nanoparticles were recovered by centrifugation (8000 rpm for 15 min) and dried under vacuum at 60 °C for 8 h, resulting in a dark powder. They were labeled as sample S.

**Figure 1:**
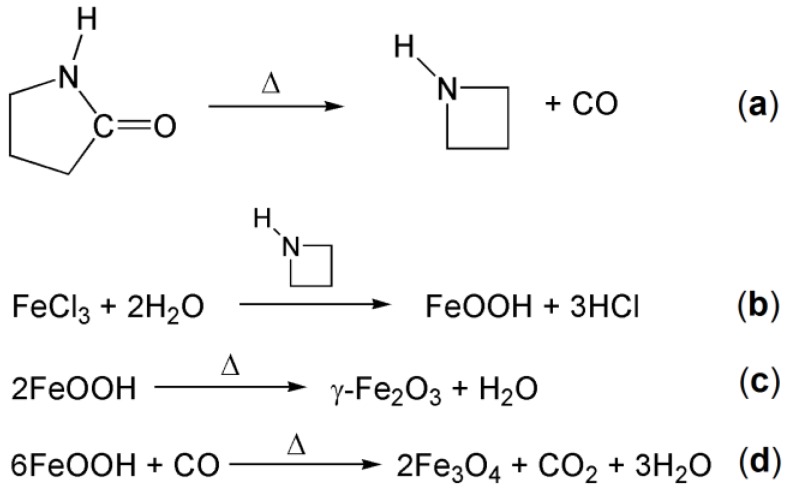
Chemical reactions involved in the synthesis of the iron oxide nanoparticles. See text for explanation.

The employed synthesis starts from iron(III) ions which can be converted to FeOOH by reaction with the hydration water molecules catalyzed by the azetidine generated in situ as schematized in Figure 1 (reactions a and b) [19]. The ferric oxide hydroxide can subsequently undergo dehydration to form γ-Fe_2_O_3_ (maghemite) and/or dehydration and partial reduction by carbon monoxide to form Fe_3_O_4_ (magnetite) (Figure 1, reactions c and d). The relative amount of the two species probably depends on the reaction temperature.

**Figure 2:**
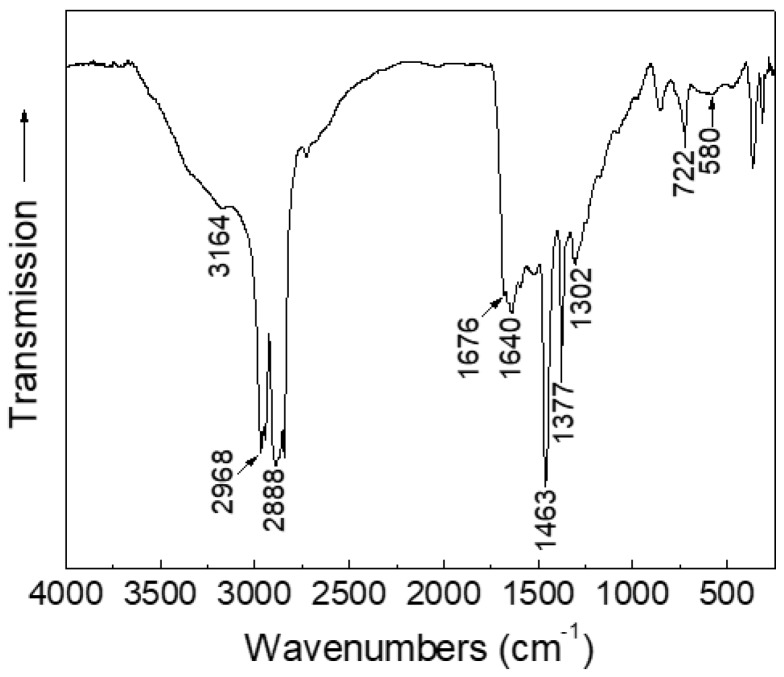
Fourier transform infrared spectroscopy (FT-IR) spectrum of the as-prepared nanoparticles (sample S). The tagged bands are discussed in the text.

The presence of 2-pyrrolidone in sample S was assessed by performing a Fourier transform infrared spectroscopy (FT-IR) analysis, as shown in Figure 2. The spectrum shows several absorptions in the investigated 4000–250 cm^−1^ range, corresponding to the vibrational modes of 2-pyrrolidone [20,22]. In particular, the bands related to the N–H bond stretching (3164 cm^−1^), to the asymmetric and symmetric CH_2_ stretching (2968 and 2888 cm^−1^, respectively), to the C=O bond stretching (1676–1640 cm^−1^), to CH deformation (1463 and 1377 cm^−1^), to CCN stretching (1302 cm^−1^), and to the N–H bond bending (722 cm^−1^) are all shifted to lower frequencies as compared to those of free 2-pyrrolidone [22]. The effect is ascribable to the coordination to iron centers at the surface of the nanoparticles, as observed in the case of poly[*N*-vinyl-2-pyrrolidone] coated nanoparticles [23] and confirms the presence of 2-pyrrolidone as capping agent. The absorption visible at ~580 cm^−1^ is not characteristic of 2-pyrollidone, but it is due to the iron oxide Fe–O bond (Figure 2).

**Figure 3:**
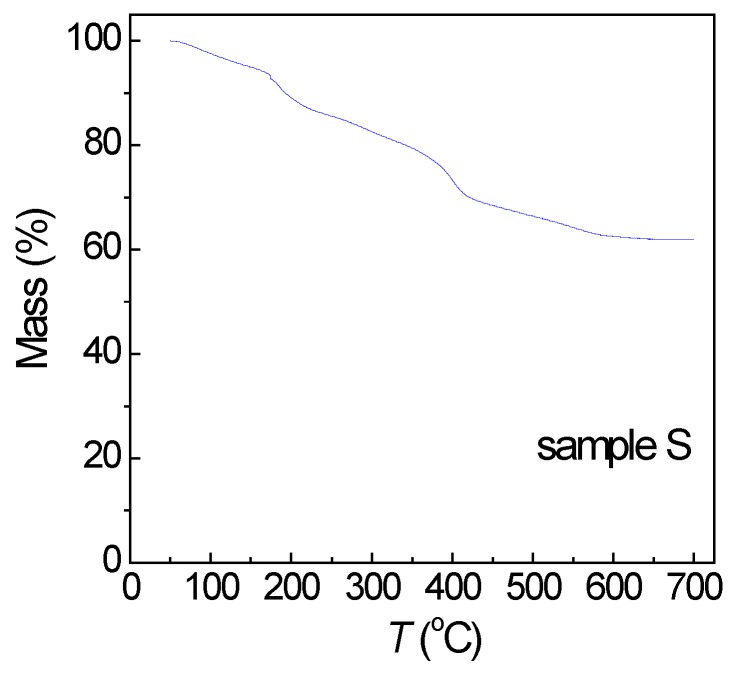
Mass loss vs. temperature (*T*) for the as-prepared iron oxide nanoparticles (sample S).

Moreover, a thermogravimetric analysis (TGA) was conducted on sample S, in nitrogen purge gas from 50 to 700 °C (heating rate = 10 °C/min). The mass loss, ascribed to the decomposition of 2-pyrrolidone and the subsequent desorption of the decomposition residues, was of ~38% (Figure 3).

Then, the as-prepared nanoparticles were dispersed in water (sample SW) and in PEG (sample SP) with a 10 mg/mL concentration.

### 2.2. Characterization Techniques

The infrared spectra were taken on a Perkin-Elmer Spectrum 100 FT-IR spectrophotometer; solid samples were dispersed in Nujol mull, whereas fluid samples were analyzed as prepared; all of the samples were cast between KBr windows for the measurement.

The morphology of the samples was investigated by transmission electron microscopy (TEM) analysis using a TECNAI FEI G2 microscope. Usually, in order to perform TEM analysis on dried nanoparticles, they must be dispersed in a liquid medium and then a drop of the suspension is deposited on a TEM grid. In our case, TEM observations were carried out directly on the ferrofluids, namely on samples SW and SP after being diluted with deionized water.

A Mössbauer spectroscopy analysis was carried out on the as-prepared nanoparticles (sample S) in a standard transmission geometry using a ^57^Co source in a Rh matrix. The spectrometer was equipped with a superconducting magnet. A high-purity iron foil was used for calibration and isomer shifts were calculated with respect to this reference sample.

The magnetic properties of the samples were studied with a Quantum Design superconducting quantum interference device (SQUID) magnetometer, operating in the 5–300 K temperature range (maximum applied field 50 kOe). The magnetic moment of the samples was measured as a function of field and of temperature.

Viscosity measurements were performed at room temperature with an Anton Paar Dynamic shear rheometer, model MCR101.

The heating capacity of SW and SP in a time-varying magnetic field was tested using a custom-made apparatus equipped with a 7-turns inductor supplied by an EASYHEAT 10.0 kW (Ambrell) generator, as described elsewhere [24]; the temperature was measured by means of an Optocom OPT-2 fiber optic thermometer.

## 3. Results and Discussion

### 3.1. Morphology and Composition

**Figure 4:**
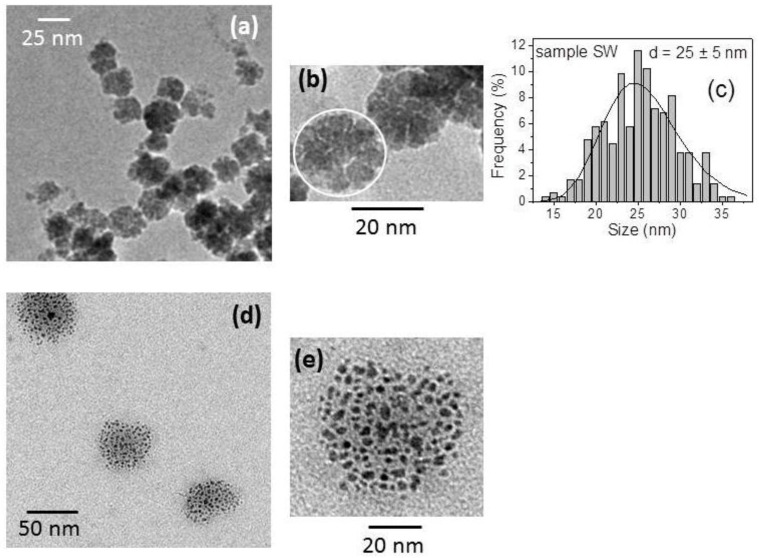
(**a**,**b**) Transmission electron microscopy (TEM) images of the as-prepared magnetic nanoparticles dispersed in water (sample SW); in (**b**) the white circle highlights one nanoflower (NF) structure and in (**c**) the size distribution of the NFs is shown (the straight line represents a best fit performed with a lognormal distribution function; d is the mean value of the distribution). (**d**,**e**) TEM images of the magnetic nanoparticles dispersed in polyethylene glycol 400 (PEG) (sample SP).

Figure 4a,b are TEM images of sample SW. The magnetic nanoparticles exhibit a rounded profile and are arranged in characteristic flower-shaped aggregates, which we indicate as nanoflowers (NFs). Several TEM images of sample SW were recorded and analyzed with the ImageJ software, so as to compute the NFs size distribution that is presented in Figure 4c (about 300 NFs were considered). These data were then fitted to a lognormal distribution function, and the calculation yielded a mean value d ~25 nm and a standard deviation of ~5 nm.

TEM images of sample SP are shown in Figure 4d,e. The arrangement of the nanoparticles is strongly different when compared to that observed in SW. Isolated groups of nanoparticles are visible, which very likely correspond to the starting NFs. However, the nanoparticles now appear quite separated so that their configuration can hardly be still described as a NF. One can distinguish different black elements with a size ranging between 5 and 10 nm, presumably corresponding to individual nanoparticles or small clusters of 2–3 nanoparticles.

We propose that the NFs form during the synthesis process since the nanoparticles are held together by strong hydrogen bonding interaction between the 2-pyrrolidone molecules bound to their surface. In fact, it is known that pure primary or secondary amides, such as 2-pyrrolidone, tend to self-associate forming dimers and higher oligomers [25]. The ability of the solvent oligomers to coordinate more nucleation centers in close proximity, during the development of the magnetic phase, could favor the formation of the NFs.

The fact that the NF structure is less stable in PEG than in water can be ascribed to the presence of the cyclic amide ligands as binders. In fact, 2-pyrrolidone is a polar molecule that coats the external surface of the nanoparticles—as suggested by the FT-IR analysis (Figure 2)—thus favoring their dispersion in polar fluids. Water and PEG are polar fluids, but the steric hindrance of their molecules is markedly different because water is a small molecule, whilst PEG is a linear polymer, H–(O–CH_2_–CH_2_)*_n_*–OH (in this case *n* ~ 9), which is expected to fold and reach a size in the 2–3 nm range [26]. We propose that, in sample SP, the large PEG molecules permeate the NFs, thus causing their expansion. On the contrary, in sample SW, the small water molecules can interact with the 2-pyrrolidone molecules on the different nanoparticles without disrupting their aggregated form.

**Figure 5:**
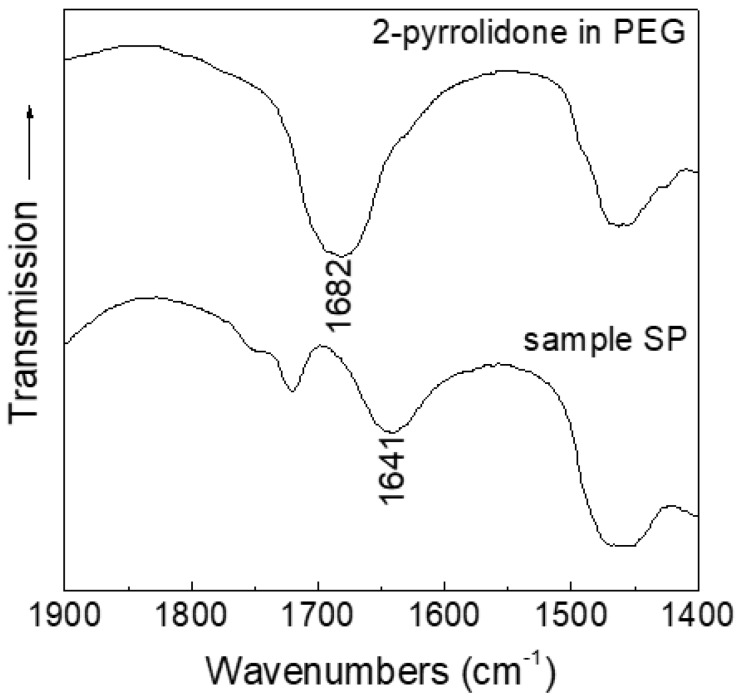
FT-IR spectra of sample SP (**bottom**) and pure 2-pyrrolidone in PEG (**top**). The tagged bands are discussed in the text.

To confirm the effect of PEG as solvent and dismiss its possible involvement as a ligand on the surface of the nanoparticles, we performed a FT-IR analysis on sample SP and, for comparison, on pure 2-pyrrolidone in PEG (Figure 5). As previously stated (Section 2.1), the shift in the C=O bond stretching can give information on the coordination of 2-pyrrolidone to the nanoparticles. The spectra reveal that in sample SP no free 2-pyrrolidone is present (the C=O stretching is shifted to 1641 cm^−1^, and the band at 1682 cm^−1^ of free ligand is not observed), indicating that no substitution of the ligands on the surface of the nanoparticles by PEG has occurred upon the dispersion in the fluid. This confirms the hypothesis that 2-pyrrolidone is strongly bound to the surface of the nanoparticles, even after dispersion in the liquid medium, and that the expansion of the NFs can be mainly ascribed to the disruption of hydrogen bonds between the nanoparticles.

Hence, the particular aggregation state of the nanoparticles as well as the possibility of disaggregating the NFs, through the interaction with the carrier fluid, are inherent to the employed synthesis method. For instance, the maghemite flower-like nanoparticles, produced by coprecipitation in form of single crystals by Hugounenq et al. [27], certainly are not expected to break up into small components whatever is the fluid in which they are dispersed.

**Figure 6:**
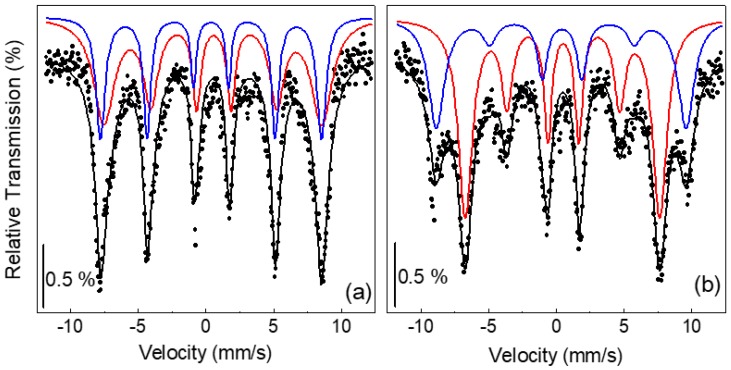
Mössbauer spectra (full symbols) recorded at *T* = 4.2 K on sample S in (**a**) B_appl_ = 0 T and (**b**) *B*_appl_ = 8 T. The black line is the fitting curve; blue and red lines are the A and B sextets, respectively.

In order to gain further structural and compositional information, the as-prepared nanoparticles (sample S) were investigated by Mössbauer spectroscopy. To reduce thermally induced magnetic relaxation effects due to the small nanoparticle size, the analysis was carried out at *T* = 4.2 K. Spectra were collected both in zero field and in a static magnetic induction field *B*_appl_ = 8 T applied along the propagation direction of the γ-rays. They are shown in Figure 6.

It is worth recalling that stoichiometric magnetite (Fe_3_O_4_) and maghemite (γ-Fe_2_O_3_) are ferrimagnetic materials with spinel structure, which implies that the iron ions occupy two different crystallographic positions, usually identified as tetrahedral (A) and octahedral (B) sites. The interaction between A and B sites is antiferromagnetic in nature. In particular, magnetite is an inverse spinel, being the divalent ions Fe^2+^ placed in the B sites and the trivalent ions Fe^3+^ equally distributed in the A and B sites. One magnetite cell contains eight units [Fe^3+^]_A_ [Fe^3+^ Fe^2+^]_B_ O_4_. A net magnetic moment arises from the Fe^2+^ ions since the contributions from the Fe^3+^ ions annihilate reciprocally.

In maghemite, only Fe^3+^ ions are present and ferrimagnetism arises from an unequal distribution of these ions in A and B sites. The maghemite cell contains eight units, as described by the formula:

[Fe^3+^]_A_ [Fe^3+^_5/3_ Υ_1/3_]_B_ O_4_ (1)

where Υ is the cation vacancy.

The zero-field Mössbauer spectrum exhibits six broad lines (Figure 6a). On the contrary, in the in-field spectrum, one can distinguish the presence of two different sextets (Figure 6b). This reveals the ferrimagnetic character of the nanoparticles. In fact, as expected, an increase of the hyperfine field of A sites and a decrease of that of B sites is observed under an applied field, so that the lines 1 and 6 of the two sextets appear well resolved [28].

Hence, both spectra were least-squared fitted with two sextets of Lorentzian-shaped lines, which are also displayed in Figure 6 together with the resulting fitting curves. The sextets have a large linewidth. For the zero-field spectrum, the linewidth is 0.4 mm/s and 0.92 mm/s for the A and B sextets, respectively (the linewidth of the calibration Fe foil pattern was 0.16 mm/s). A similar broadening of the spectral lines is observed in the in-field spectrum. This effect is to be connected with a high degree of structural disorder since it indicates that not all of the Fe ions located in both the A and B sites are surrounded by an identical atomic environment, actually [29,30,31].

The fitting parameters are reported in Table 1: isomer shift IS, hyperfine field *B*_hf_ (measured without applied field) and effective hyperfine field *B*_eff_ (measured in the presence of *B*_appl_), ratio between the relative resonant area of each sextet *X*_A_/*X*_B_ (equal *f* factors have been assumed for the resonant atoms that are located in the two different configurations). The quadrupolar shift is not reported in Table 1 as it remains close to zero for both sites.

As maghemite can result from the oxidation of magnetite, ferrimagnetic iron oxide nanoparticles are often reported to consist of a mix of these two phases, which are very difficult to distinguish. In our case too, the Mössbauer analysis is not able to assess unambiguously the chemical composition of the nanoparticles. The values of *B*_hf_ for A and B sites are almost equal, as expected for γ-Fe_2_O_3_ [28]. However, since the hyperfine field in nanostructured spinel oxides may be somewhat altered as compared to the bulk phases [31,32,33,34], we cannot precisely discriminate between γ-Fe_2_O_3_ and Fe_3_O_4_ on the basis of *B*_hf_.

However, the quite low IS values, varying between 0.40 and 0.57 mm/s, support a prevalent presence of Fe^3+^ ions [35,36,37]. Moreover, the X_A_/X_B_ ratio is ~0.57, which is very close to the theoretical value for γ-Fe_2_O_3_ (0.6). Hence, based on the hyperfine parameters, we are inclined to think that the nanoparticles consist mainly of maghemite even if the presence of magnetite, namely of Fe^2+^ ions, cannot be totally excluded.

With reference to Figure 1, it appears that, with the adopted synthesis parameters of temperature and FeCl_3_ relative concentration, the reaction d, which would have led to the formation of magnetite, was partially inhibited. This demonstrates the versatility of the synthesis method designed by Li et al. [19] and, on the other hand, the need of carefully characterizing the nanoparticles produced in that way.

In the presence of an external magnetic field, the spins of the Fe ions are to some degree aligned by the field. In bulk maghemite the magnetization of the A sublattice is antiparallel to that of the B sublattice—i.e., the structure is collinear—and when a saturating magnetic field is applied parallel to the γ-ray direction, the area of lines 2 and 5 of the sextets reduces to zero [28]. If this does not occur, the evidence is obtained that the structure is not collinear, and, hence, even a very strong magnetic field is unable to attain a complete spin alignment. Usually, iron oxide nanoparticles show such a spin canting phenomenon, and a long-debated issue is whether it is a surface effect, involving only the spins located in a surface layer [33,38], or a finite size effect, involving also core spins [39,40].

In this respect, it should be considered that different synthesis methods and even small differences in the preparation procedures of the nanoparticles could result in a variety of sizes, shapes, and crystallinity degrees [12]. For this reason, in our opinion, the matter depends on the characteristics of the nanoparticles. In any case, spin canting is the hint of structural disorder at the surface and/or in the core of the nanoparticles, since it generally arises because of modified atomic coordination and altered superexchange bonds. The value of the effective field B_eff_ results from the vectorial sum of *B*_hf_ with *B*_appl_, according to the relation:

*B*_eff_^2^ = *B*_hf_^2^ + *B*_appl_^2^ + 2 *B*_hf_*B*_appl_ cosδ (2)

where δ is the angle between the directions of *B*_hf_ and *B*_appl_ [28].

Applying the relation (2) to the Fe ions in the A and B sites, one can calculate δ_a_ and δ_b_; the canting angles that A and B spins form with the applied field are θ_a_ = δ_a_ and θ_b_ = (180° − δ_b_) (Table 1).

### 3.2. Magnetic Properties and Heating Capacity

**Figure 7:**
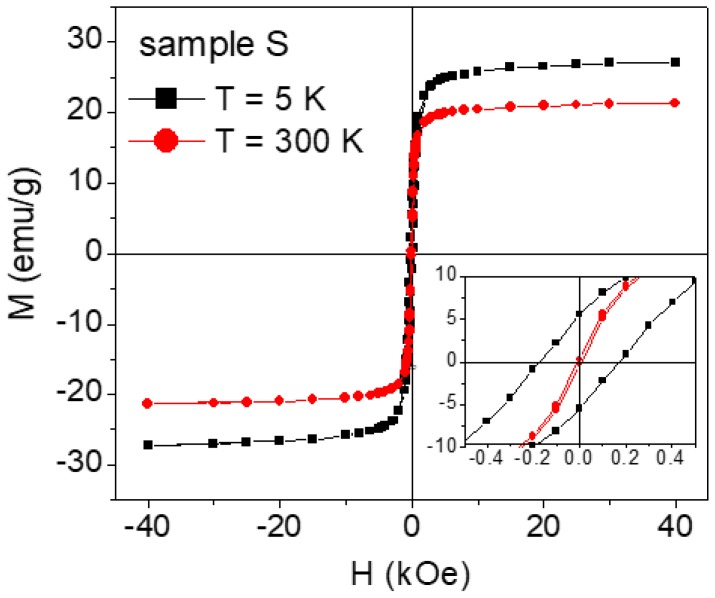
Magnetic hysteresis loops recorded on the as-prepared nanoparticles (sample S) at *T* = 5 K (black symbols) and *T* = 300 K (red symbols). The specific magnetization M, relative to the whole sample mass (including 2-pyrrolidone), is reported on the *y*-axis. The inset is an enlarged view of the central region of the loops.

Magnetic hysteresis loops measured on sample S at *T* = 5 and 300 K are shown in Figure 7. In particular, the specific magnetization (M) is reported, obtained by dividing the magnetic moment, as measured by SQUID, for the whole sample mass, which also includes the mass of 2-pyrrolidone. The saturation magnetization, extrapolated from the loop at *T* = 5 K for 1/H tending to zero, is *M*_S_ ~ 28 emu/g. To obtain M_S_ of the iron oxide phase only, we must subtract the mass of 2-pyrrolidone, estimated by TGA (Figure 3), from the total sample mass. Hence *M*_S_ ~ 45 emu/g, substantially lower than the saturation magnetization value expected for bulk maghemite (~83 emu/g). This magnetization reduction is certainly connected to the spin canting effect, as revealed by the Mössbauer analysis.

Based on the values of θ_a_ and θ_b_ (Table 1), we can calculate the expected value of *M*_S_ for our nanoparticles, assuming that they are totally made of maghemite. With reference to formula (1), it is known that the filled A site has moment μ_a_ = 4.18 μ_B_ (μ_B_ = Bohr magneton) and the 5/3 filled B sites have moments μ_b_ = 4.41 μ_B_ [41]. Hence, the effective moment per unit (1) can be calculated through the relation:

μ_eff_ = −μ_a_ cosθ_a_ + 5/3 μ_b_ cosθ_b_ (3)

We obtain that μ_eff_ ~ 1.83 μ_B_. In the case of collinear maghemite (i.e., θ_a_ = θ_b_ = 0°), the moment per unit is 3.17 μ_B_. Therefore, the spin canting effect produces a magnetization reduction of ~42%. Accordingly, the specific magnetization *M*_S_ is expected to decrease from the nominal value of ~83 down to ~48 emu/g, in excellent agreement with the experimental value. It is to be noted that Equation (3) implies that all the maghemite spins are canted. The good agreement between the experimental and calculated M_S_ values indicates that indeed this must be the case of our nanoparticles.

In sample S, the coercivity *H*_C_ ~ 170 Oe at *T* = 5 K and it decreases down to ~6 Oe at *T* = 300 K (Figure 7). The remanent magnetization *M*_r_ also shows a strong thermal dependence: the ratio between the remanence and the magnetization at *H* = 40 kOe is ~0.2 at 5 K and ~0.01 at 300 K.

As for samples SW and SP, in order to obtain *M*_S_, the measured hysteresis loops were corrected by subtracting the diamagnetic signals deriving from the carrier fluid and from the special holder for liquids. In both of the samples, the value of M_S_ was consistent with that expected when considering the saturation magnetization of the as-prepared nanoparticles and their concentration; at *T* = 250 K, no hysteresis was observed, namely *H*_C_ and *M*_r_ were equal to zero (to impede any movement of the nanoparticles during the SQUID analysis, the measurements on SW and SP were performed at temperature lower than 250 K, at which the carrier fluids were in the frozen solid state).

**Figure 8:**
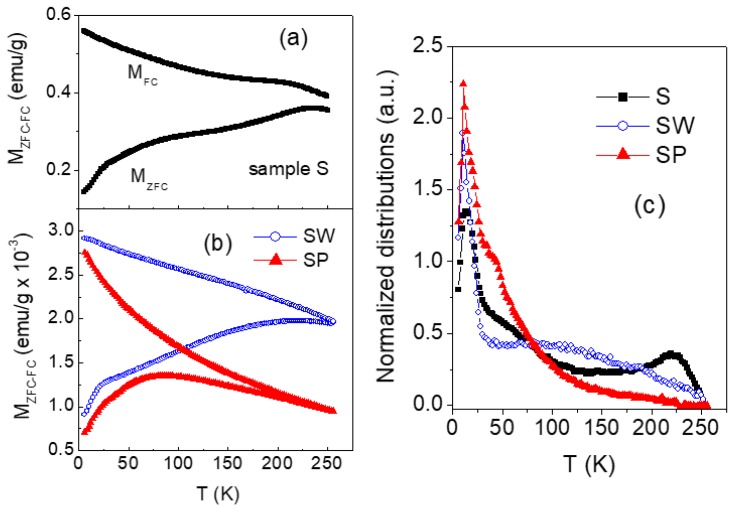
(**a**,**b**) Magnetization measured in ZFC mode (lower branches) and FC mode (upper branches) for increasing temperature (*T*) in *H*_appl_ = 10 Oe on samples S, SW and SP. (**c**) Temperature derivative [−d(*M*_FC_−*M*_ZFC_)/d*T*] of the difference between field-cooled and zero-field-cooled magnetizations for samples S, SW, and SP (the curves are normalized to their area).

The magnetothermal behavior of samples S, SW and SP was investigated by performing magnetization measurement as a function of increasing temperature (heating rate 2 K/min) in a static magnetic field *H*_appl_ = 10 Oe, after cooling the sample from room temperature down to *T* = 5 K without applied field (zero-field-cooling, ZFC) and in the presence of *H*_appl_ (field-cooling, FC). The results are shown in Figure 8a,b.

Magnetic irreversibility (difference between *M*_FC_ and *M*_ZFC_) is observed in the as-prepared nanoparticles in all the spanned temperature range. This indicates that thermally induced magnetic relaxation processes occur, but that the full superparamagnetic state of the nanoparticles assembly is not achieved up to *T* = 300 K. This behavior is consistent with the strong reduction of *H*_C_ and *M*_r_, passing from *T* = 5 K to *T* = 300 K, where small but non-zero values are measured (Figure 7). A quite similar magnetic irreversibility effect is found in SW, whereas in SP the ZFC and FC branches are superposed for *T* > 200 K.

For an assembly of non-interacting magnetic nanoparticles, the temperature derivative of the difference (*M*_FC_ − *M*_ZFC_) is a figure of the distribution of anisotropy energy barriers for magnetic moment reversal, which substantially reflects the size distribution [16]. In fact, the anisotropy energy barrier associated to one nanoparticle is given by *KV*, where *K* is the magnetic anisotropy coefficient and *V* is the volume. In our case, it is to be expected that the magnetothermal behavior of the nanoparticles is affected by magnetic interactions, mainly dipolar in type because of the presence of the surfactant that prevents a direct exchange coupling. In this case, the derivative of (*M*_FC_ − *M*_ZFC_) provides qualitative though valuable information on the distribution of effective anisotropy energy barriers [42,43], which can be associated to a distribution of effective magnetic sizes.

For our samples, the curves of [−d(*M*_FC_−*M*_ZFC_)/d*T*] are shown in Figure 8c (normalized to their area). The distribution of the as-prepared nanoparticles (sample S) extends over the whole investigated temperature range and features a narrow peak at *T* ~ 10 K and a second one at *T* ~ 220 K.

Passing to the distributions of SW and SP, the peak at ~10 K is still well visible, whereas a very different profile is observed at higher temperature. In fact, regarding SW, a very broad peak at *T* ~ 100 K is visible; as for SP, just a shoulder, followed by a broad tail, appears on the right side of the low-temperature peak.

If we describe the relaxation process using the Néel expression for the relaxation time, a rough estimation of the magnetic size that is associated to the two peaks in the distribution of sample S can be done through the well-known relation *KV* = 25 k_B_*T*_p_, where *K* is the magnetic anisotropy coefficient of the nanoparticle, *V* is the volume, k_B_ is the Boltzmann constant, and *T*_p_ is the temperature of the peak [16,43,44]. In our case, the anisotropy is unknown, but we can tentatively assign to *K* the value of bulk maghemite (5 × 10^4^ erg/cm^3^). Hence, we obtain ~11 nm and ~30 nm for the first and second peak, respectively. These values are close to the sizes of the separated nanoparticles and of the NFs, as indicted by TEM (Figure 4). Hence, we infer that the peak at ~10 K is to be associated to a fraction of almost isolated or weakly interacting nanoparticles, whereas the energy barriers at higher temperature, and in particular the peak at ~220 K, arise because of the tendency of the nanoparticles to arrange in form of NFs.

The fact that, passing from sample S to SW and to SP, the distribution shrinks and shifts towards low temperature values indicates that the presence of the fluid reduces the strength of the interparticle magnetic interactions, which results in magnetic aggregates with a smaller effective magnetic size. This effect is especially remarkable in sample SP, in agreement with the TEM analysis that indicates a substantial disaggregation of the NF structures (Figure 4d,e). As for sample SW, the broad peak centered at *T* ~ 100 K (Figure 8c) corresponds to an average effective magnetic size of ~24 nm, consistent with the dimension of the aggregates in Figure 4a,b. The fact that the first peak at *T* ~ 10 K is visible in the three distributions and that its intensity is larger in SP than in the other samples supports this description.

A significant example, demonstrating to what extent the different arrangement of the nanoparticles in the carrier fluids may affect the physical properties, is provided by the study of the magnetic heating capacity, tested by exposing 2 mL of SW and SP to an alternating magnetic field for 300 s.

**Figure 9:**
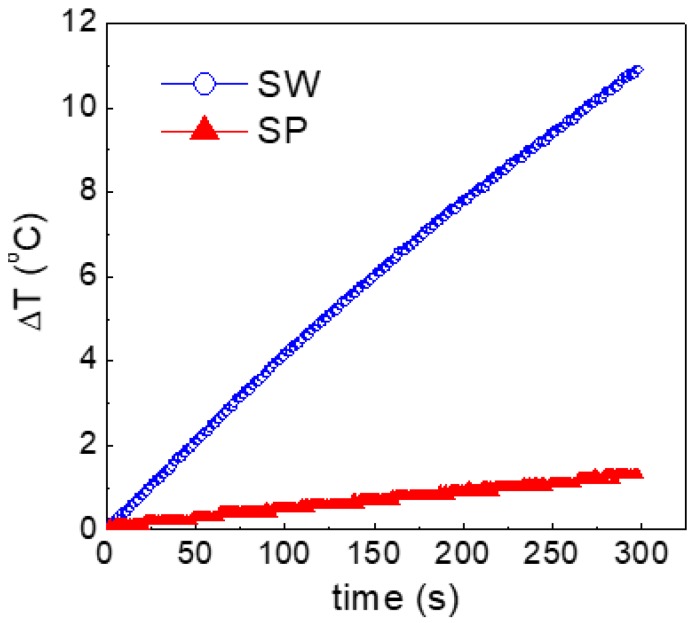
Heating curves for samples SW and SP in an alternating magnetic field (170 Oe in amplitude at frequency 177 kHz).

The field amplitude was of 170 Oe and the frequency 177 kHz. These parameters were chosen because, for prospective in-vivo biomedical applications, their product (~3 × 10^7^ Oe Hz) follows the safety guideline to avoid detrimental effects of eddy currents on healthy tissues [45]. The results are shown in Figure 9: the temperature increase Δ*T* is ~11 °C for sample SW and ~1.5 °C for sample SP.

It is worth remarking that the same amount of nanoparticles was present in the two measured samples and that the density and specific heat values for water and PEG are 1 g/cm^3^ and 1 cal/g·°C for the first one and 1.1 g/cm^3^ and 0.51 cal/g·°C for the second one. As for the viscosity *η* —which is also a crucial parameter for the heating efficiency [46]—measurements carried out on SW and SP at room temperature revealed that it was similar in the two samples (*η* ~ 33 × 10^−2^ P) and close to that of PEG (reference viscosity values at 20 °C of PEG and water are *η* ~ 32 × 10^−2^ P and *η* ~ 1 × 10^−2^ P, respectively), and that it did not depend on the applied shear rate (i.e., the ferrofluids showed a Newtonian behavior). It is worth noticing that this result appears to be consistent with our picture regarding the arrangment of the nanoparticles in the two fluids. In SP, the nanoparticles are separated and do not offer resistance to the flow strong enough to substantially alter the viscosity of PEG. On the contrary, the remarkable increase of η passing from water to SW indicates that the large NFs offer a strong resistance to the flow, although the Newtonian character of the ferrofluid is preserved. This interesting effect may be tentatively related to the presence of ordered and tightly bound 2-pyrrolidone molecules on the NFs structure, which interact strongly with the aqueous medium by hydrogen bonding, whereas, in PEG, the interaction is of lower extent and is partially disrupted. The phenomenon will deserve further attention.

Coming back to the heating capacity, based on the above considerations, the result in Figure 9 is to be entirely ascribed to the different arrangement of the nanoparticles in the two fluids.

It is well known that the amount of heat released by magnetic nanoparticles subjected to the varying magnetic field is determined by different parameters, e.g., the saturation magnetization, the magnetic anisotropy and the size [46,47]. A crucial role is also played by interparticle dipolar interactions, although their effects on the heating efficiency are still a controversial issue. Dipolar interactions are reported to have a detrimental effect because they diminish the effective field acting on each particle [48]. Moreover, in the case of aggregates, large hydrodynamic sizes slow down the Brownian relaxation, thus reducing the heating efficiency [49].

On the other hand, several studies predict [50,51] and experimentally demonstrate [52] that dipolar interactions result in a substantial increase of the magneto-thermal stability of the nanoparticles and in enhanced hysteresis losses, and, hence, in improved heating performances. The results in Figure 9 support this last scenario, namely indicate that, in the adopted experimental conditions, a larger amount of heat is released by the nanoparticles in form of NFs.

The noteworthy conclusion is that the heating capacity of the produced iron oxide nanoparticles can be tuned by properly selecting the fluid in which they are dispersed, which determines their aggregation state.

## 4. Conclusions

Magnetic nanoparticles, with mean size between 5 and 10 nm, were produced by thermal decomposition of iron(III) chloride hexahydrate (FeCl_3_·6H_2_O) in 2-pyrrolidone and successively dispersed in water and PEG. Experimental techniques for chemical, structural, and magnetic characterization were synergically employed to study the nanoparticles in the as-prepared state and in the form of ferrofluids.

With the reported synthesis, parameters of temperature and FeCl_3_ relative concentration, nanoparticles made of spinel iron oxides are obtained, maghemite being the predominant phase, as indicated by the Mössbauer spectroscopy analysis. The Mössbauer results for sample S are also consistent with a strong structural disorder and a marked spin canting effect, resulting in a low saturation magnetization, about half of the expected one for bulk γ-Fe_2_O_3_.

The peculiarity of the produced nanoparticles is that they form NF aggregates—having a mean size of ~25 nm, as shown by TEM—which are substantially stable in water and tend to disaggregate in PEG. The particular aggregation state of the nanoparticles and their behavior in the carrier fluids can be ultimately connected to the presence of the strong polar 2-pyrrolidone at their surface, as revealed by FT-IR and TGA measurements. Hence, although one may object that the structural and magnetic properties of the nanoparticles prepared by the adopted synthesis method are not optimal for applications, it should be considered that it is just the synthesis method that determines the NF configuration of the nanoparticles and the interesting way they re-arrange in the carrier fluids. As indicated by the analysis of the magnetothermal behavior of samples SW and SP, the different state of aggregation of the nanoparticles in water and PEG directly affects the strength of interparticle magnetic interactions, which can be crucial in determining the final performances of the ferrofluids. As a support to this last statement, we have compared the magnetic heating capacity of SW and SP, definitely higher for the former.

Our study paves the way to the possibility of tailoring the properties of ferrofluids by controlling the aggregation state, and hence the degree of magnetic coupling, of properly engineered nanoparticles through their interplay with the carrier fluid.

## Figures and Tables

**Table 1:** Parameters deduced from Mössbauer spectra measured in *B*_appl_ = 0 and 8 T: isomer shift (IS), hyperfine field in no applied field (*B*_hf_), hyperfine field in applied field (*B*_eff_); canting angle θ for A and B sites, ratio between the relative resonant area of the A and B sextets (*X*_A_/*X*_B_).

*B*_appl_ (T)	Site	IS (mm/s)	*B*_hf_ (T)	*B*_eff_ (T)	θ (°)	*X*_A_/*X*_B_
0	Fe(A)	0.40 ± 0.02	50.7 ± 0.1	-	-	0.57 ± 0.03
	Fe(B)	0.57 ± 0.03	50.0 ± 0.1	-	-	
8	Fe(A)	0.41 ± 0.01	-	57.4 ± 0.1	36 ± 2	0.57 ± 0.02
	Fe(B)	0.51 ± 0.03	-	44.7 ± 0.1	45 ± 2	
